# Impulsivity and Empathy in Dating Violence among a Sample of College Females

**DOI:** 10.3390/bs10070117

**Published:** 2020-07-20

**Authors:** Arta Dodaj, Kristina Sesar, Nataša Šimić

**Affiliations:** 1 Department of Psychology, University of Zadar, 23000 Zadar, Croatia; 2 Department of Psychology, University of Mostar, 88000 Mostar, Bosnia & Herzegovina; kristina.sesar@ff.sum.ba

**Keywords:** dating violence, college females, impulsivity, empathy

## Abstract

The predictive factors of violence between married couples or adolescents are well-known. However, less is known about the factors relating to intimate violence among college students. This study examined sociodemographic variables (age, duration of relationship, and relationship satisfaction), impulsivity, and empathy as predictors of dating violence, using data from 474 female college students from the University of Mostar, Bosnia and Herzegovina. The sample completed online the Conflict Tactics Scale 2 Short Form, the Interpersonal Reactivity Index, and the Short Impulsive Behavior Scale. The results indicated a higher prevalence of victimization than perpetration for psychological aggression. The obtained data showed that younger women and those in longer relationships, as well as those unsatisfied with their relationship, are more prone to experience psychological victimization or perpetration. Relationship satisfaction was also shown to be a predictor of physical perpetration causing injury. Impulsivity facets were found to have a differential weight in explaining dating violence. Empathy was shown to be a significant predictor of dating victimization, specifically “perspective taking” for psychological victimization and empathic concern for sexual victimization. These results suggest the need to develop specific interventions and prevention programs focused on relationship satisfaction, impulsivity, and empathy.

## 1. Introduction

Dating violence (DV) has been shown to be a widespread issue during adolescence and young adulthood [1]. It involves intentional acts of sexual, physical, or psychological abuse by one member of a dating partnership towards the other [2], and these different types of violence very often happen together [3]. DV occurs in the context of an intimate relationship between adolescents and young individuals, with varying degrees of formality [4], who do not live together and have no children or binding legal or economic ties [5].

The rate of DV varies according to the inclusion criteria used and differences in conceptual and methodological approach [6]. There are high variations in the prevalence in the student population, ranging from 5.5% to 65.6% [7,8,9,10]. Physical violence has been found in 17% to 45% of college relationships [11], sexual violence in 15% to 20%, and psychological/emotional violence in 50% to 80% [12,13,14,15]. The evidence regarding sex differences is mixed. There have been numerous studies in the past 40 years [11,16,17,18,19,20] that found a high prevalence of victimization of young women and perpetration by young males. Some recent studies note that they also show high perpetration rates and high male victimization rates [21,22,23,24,25]. In understanding the risks, relationship dynamics are also important. Couples who had poorer relationship quality at the beginning of young adulthood were more likely to experience current physical and psychological abuse [26]. O’Leary et al. [27] stated that individuals dissatisfied with a relationship are more prone to perpetration than those who are satisfied.

One of the most important individual determinants in the context of social behavior is empathy [28]. Davis [29] argued that empathy is best understood as a multidimensional dispositional construct that is measured by taking both cognitive and affective components into consideration. His concept is comprised of four dimensions: perspective taking (the ability to understand the cognitive perspective of another); empathic concern (the affective, emotional response to another’s experience); fantasy (the tendency to fantasize or place oneself in fictitious situations); and personal distress (the level of anxiety in tense interpersonal situations). The author [29] developed a measure of dispositional (trait) empathy known as the Interpersonal Reactivity Index (IRI), consisting of these four dimensions. Although there is no agreement about the most appropriate definition and measure of empathy, Davis’s measure has been used and validated on many different populations over the years.

The existence of empathy has been related with prosocial helping behaviors [28], while its lack has been related to aggressive behavior [30]. Researchers have found that, for nonviolent couples, empathy plays a significant role in navigating interpersonal situations, and is positively associated with the quality of the relationship [31]. In the area of empathy and offending, one meta-analytic study found that low cognitive empathy was strongly associated with offending [32]. Others reported that the offender’s personality, such as a low level of anger control and empathy, was related to aggression and violence toward others [33]. Other authors have also suggested that the cognitive component of empathy (i.e., perspective taking) significantly inhibits interpersonal aggression in an experimental setting [34], and as well as that, low empathy is associated with dating and sexual violence among adolescents and male college students [35].

Psychological research has also shown differential expression of empathy based on gender [36]. Although women are much more prone to an affective response based on empathy than men, there are no significant gender differences in terms of cognitive empathy [36]. There is evidence that does not support the role of gender in moderation of the relationship between empathy and violence [37]. Whether empathy predicts DV differently between men and women is still unclear. The relationship between empathy and violence has primarily been examined in men (e.g., prison inmates, perpetrators of serious violent crimes, externalizing behavior in adolescent males) or in children. Nonetheless, literature findings on marital violence show that similar variables are related to partner violence among men and women [38].

Impulsivity is a personality trait that can be operationalized as a lack of self-reflection and planning, with rapid and careless decision-making, decreased sensitivity to negative consequences, and a lack of regard for long-term consequences [39,40]. Specifically, a large amount of research has been shown to suffer from problems related to the variability in how the researchers conceptualize the variable of impulsivity, which contributes to significant inconsistencies between measures. To provide some consistency, Whiteside and Lynam [41] used a Five Factor Personality Model [42] to organize traits and behaviors into the ten most commonly used impulsivity measures. The authors obtained four common factors across the different measures, including urgency, lack of premeditation, lack of perseverance, and sensation seeking. Urgency represents the tendency to experience strong impulses in reaction to a negative impact [41]. Premeditation reflects the tendency to reflect and consider the possible consequences of an action before engaging in it [41]. Perseverance refers to one’s ability to focus even on tedious tasks and engage in self-discipline [41]. Finally, sensation seeking reflects the openness and enjoyment that is derived from activities that are exciting, new, or dangerous [41]. Taken together, highly impulsive individuals engage in impulsive behaviors to reduce negative emotional states. Hence, they have difficulty maintaining focus and engage in exciting or dangerous behaviors, all without considering the consequences of those behaviors.

Impulsivity has been found to be linked with aggressive behavior [43], including the perpetration of intimate partner violence (IPV) [44,45,46]. It is especially pronounced in relation to perpetration, because it has been characterized as the inability to regulate one’s behavior in response to negative affect [47]. As such, high impulsivity serves as a factor that exacerbates a person’s likelihood for aggression, while low impulsivity or high self-control serves as a factor that inhibits aggression. It may be that impulsive individuals turn to violence as a way of coping with relationship conflict and stress [45].

This paper aimed to fill these gaps by investigating the relationship between relation satisfaction, dispositional empathy, impulsivity, and violence in dating relationships between college students. According to our knowledge, there have been no previous studies about these predictors of DV in Bosnia and Herzegovina. There is only one study that has examined prediction of DV using selected variables on a sample of young people from Croatia, a country that is culturally similar, as well as geographically near, to Bosnia and Herzegovina. The authors Ajduković et al. [48] examined the relative contribution of selected school variables (school performance and type of school) and sociopsychological variables (beliefs about a quality relationship, the importance of rights in a relationship) to an explanation of committing and experiencing violence. They found that significant predictors of experience of DV were gender and false beliefs about a quality relationship, while committing DV was predicted by gender, false beliefs about relationships, poor school performance, and the importance of rights in a relationship. Although these findings revealed important issues regarding the prediction of DV, they did not provide insight into the variables of interest in this study.

Therefore, the first aim of this study was to determine the prevalence of DV among female college students. Based on the previous results (e.g., [49,50]), we tested the hypothesis that distinct types will be evident and that victimization would be more prevalent than perpetration. More specifically, taking into account that psychological DV is most common type (e.g., [13,15,51]) led us to expect its higher prevalence. The second aim was to examine the association between satisfaction in a relationship, and the empathy and impulsivity and DV. As previous studies indicated [52,53,54,55,56], we hypothesized that satisfaction and empathy would be negatively correlated with DV, and higher levels of empathy and relationship satisfaction associated with lower levels of victimization and perpetration. Regarding impulsivity and various forms of aggressive behavior [43,44,45,46], positive relationships with victimization and perpetration were also hypothesized.

## 2. Materials and Methods

### 2.1. Participants

The participants were 474 female college students attending the University of Mostar in Bosnia and Herzegovina. According to the data obtained in the 2018–2019 academic year, there were 9418 students attending this university, of which 6162 were female students [57]. We invited 2250 female students who were in a committed relationship to participate in the study. The response rate was quite low, but studies including e-mail surveys usually tend to have lower rates [58]. The participants’ ages ranged from 19 to 29 years (M = 22.232, SD = 2.053). They identified their ethnicity as Croatian (96.202%), Bosnian (2.742%), and Serbian (1.054%). The majority of the participants were Catholic (N = 432; 91.139%), 2.109% (N = 10) considered themselves to be Muslim, 1.054% (N = 5) were Orthodox, while the remaining stated “other” (N = 15, 3.164%) or did not want to report their religion (N = 12; 2.531%). All participants had been in a committed intimate relationship in the year before data collection. The relationships ranged in length from three months to eight years, with an average duration of 32.394 months.

### 2.2. Measures

#### 2.2.1. Dating Violence

Dating violence was measured using the Conflict Tactics Scale 2 Short Form (CTS2S) [59]. This is the most commonly used measure of violent behavior during conflicts in dating or marriage relationships, as well as cohabiting relationships [60]. The scale consists of five subscales: psychological aggression, physical assault, sexual coercion, injury, and negotiation. Psychological aggression assesses tactics that cause the partner psychological distress, such as verbal and nonverbal aggressive behaviors (e.g., I insulted or swore or shouted or yelled at my partner.). The subscale of physical assault assesses the frequency of use of physical aggression and tactics (e.g., I pushed, shoved, or slapped my partner.). Sexual coercion examines the frequency of behavior intended to force a partner to engage in unwanted sexual activity (e.g., My partner used force, such as hitting, holding down, or using a weapon to make me have sex.). The injury subscale examines the physical injuries caused by physical conflict (e.g., I had a sprain, bruise, or small cut, or felt pain the next day because of a fight with my partner.). The negotiation scale measures the frequency of discussion tactics used to resolve conflicts, and the level of concern showed to the partner (e.g., My partner explained his or her side or suggested a compromise in a disagreement with me.). Each subscale measures both violent acts that the participant suffered from their partner (victimization), as well as the violent behaviors that they used against their partner (perpetration). Participants were asked to assess the frequency of occurrence over the past 12 months on a scale from 0 (never in the past three months) to 6 (more than 20 times in the past three months). The present study did not include data on the negotiation subscale since it does not assess the type of violent behavior. The frequency method of scoring recommended by Straus and Douglas [59] was used. For the prevalence rate, the results were dichotomized in the response format. From the literature, we did not find standard norms or clinical cutoff values for a group of this age in CTS2; therefore, we used a cutoff point that was based on an arbitrary clinical limit of the 65% percentile. Straus and Douglas [59] showed that the CTS2S subscales have good internal consistency. The scale was translated into Croatian and used in previous research [61,62]. Cronbach’s alpha coefficients in our female college sample ranged from 0.76 to 0.92.

#### 2.2.2. Empathy

The Interpersonal Reactivity Index (IRI), developed by Davis [63], was used to assess the multidimensional component of empathy. The IRI comprised 28 items divided into four subscales: perspective taking (PT), fantasy (FS), empathic concern (EC), and personal distress (PD). The PT subscale measures the tendency to accept spontaneously the psychological views of others (e.g., I believe that there are two sides to every question and try to look at them both). The FS subscale measures the tendency to transpose oneself imaginatively into the feelings and actions of fictional characters in books, movies, and plays (e.g., After seeing a play or movie, I felt as though I were one of the characters.). Further, the EC subscale measures the tendency to experience feelings of sympathy and care for unfortunate others (e.g., I would describe myself as a pretty soft-hearted person.), while the PD subscale measures the tendency to experience feelings of personal anxiety and discomfort in a tense interpersonal environment (e.g., When I see someone who badly needs help in an emergency, I go to pieces.). Each subscale contains 7 items, to which participants answered on a 5-point Likert scale, ranging from “does not describe me well” to “describes me very well.” The IRI shows good psychometric properties [29]. The IRI has been translated and used on a Croatian sample of college students. Internal consistency is 0.84 (range 0.71–0.77 for its subscales) [64]. Similar results were found on our female college students, with lower value of Cronbach’s alpha coefficient (0.76).

#### 2.2.3. Impulsivity

Impulsivity was measured using the Short Impulsive Behavior Scale (SUPPS-P) [65]. This 20-item scale contains five subscales, with 4 items per subscale, each tapping a separate facet of impulsivity. The negative urgency (NU) subscale measures the tendency to behave rashly under extreme negative emotions (e.g., Sometimes when I feel bad, I can’t seem to stop what I am doing, even though it is making me feel worse.). The positive urgency (PU) subscale assesses the tendency to behave rashly under extreme positive emotions (e.g., When I am in great mood, I tend to get into situations that could cause me problems.). The sensation seeking (SS) subscale measures the tendency to seek a novel and thrilling experience (e.g., I would enjoy the sensation of skiing very fast down a high mountain slope.). The lack of perseverance (PE) assesses the inability to focus on a task (e.g., Once I get going on something I hate to stop). Further, the lack of premeditation (PR) taps the tendency to behave without thinking (e.g., I like to stop and think things over before I do them.). Participants rate their level of agreement with each item on a 4 point Likert scale, ranging from “disagree strongly“ to “agree strongly“. The authors of the original scale reported reliability for each of the subscales (0.74–0.85) [65]. In our study, Cronbach’s alpha coefficient for SS subscale was low (0.65), while for other subscales it was acceptable in range from 0.70 to 0.78.

### 2.3. Procedure

Students enrolled at the University of Mostar were sent personal e-mails with an invitation to be involved in the study. Participants were included if they were in a committed intimate relationship during the study of a duration of three months or more, and if they were not older than 30 years. After a brief introduction and giving written consent to participation in the study, the participants could complete the above-mentioned questionnaires. As an incentive to participation they could ask for a brief report on the study data. The total administration time needed to complete questionnaires was approximately 15 min. The study was approved by the ethics committees of the Department of Psychology, University of Mostar, code 04/I-978/20.

### 2.4. Statistical Analysis

Analysis of descriptive data was carried out to provide the basic sample characteristics and the prevalence of DV types. After that, we ran a Chi-square test to examine possible differences in the prevalence of victimization and perpetration. We then calculated zero order correlations between the demographic data, impulsiveness, empathy, and DV. The main analyses included multiple hierarchical regression equations. For each of the criteria of DV measured, separate hierarchical multiple regressions were performed. In each case, the age, the duration, and satisfaction with the relationship were entered in the 1st step; impulsivity facets were entered in the 2nd step; and empathy facets were entered in the 3rd step. Multicollinearity was tested using tolerance and VIF statistics, and was shown to be acceptable in all cases. The highest level of VIF values were 1.7, and the lowest level of tolerance values were 0.57. All multivariate outliers were dropped out from the analysis on the basis of the Mahalanobis distance and the Cook distance index.

## 3. Results

### 3.1. Prevalence Rates

As presented in Table 1, 274 (57.81%) of the participants reported engaging in at least one type of violent behavior in the previous year. Specifically, 34.81% reported experience of psychological aggression, 2.95% experienced injury, whereas less than 1% reported experience of sexual coercion (0.84%) or physical assault (0.42%). In relation to perpetration of violence, psychological aggression appeared to be the most prevalent type, where 23% admitted using this type of violent behavior. Physical assault was reported by 2.11% of the sample, whereas occurrence of violent behavior that left an injury was reported by 1.48%. Sexual coercion was the least reported type of perpetration, with a prevalence of 0.84%. Given the prevalence of victimization and perpetration for different types of DV, we tested whether there were significant differences. Using Chi-square test with the Yates correction and Bonferroni corrected *p*, differences between perpetration and victimization for all four DV types were not found (χ2 = 7.59, df = 3, *p* = 0.055, Bonferroni corrected *p* = 0.012). However, significant difference was confirmed between psychological aggression (χ2 = 11.45, df = 1, *p* < 0.001), which presented the most prevalent type. The occurrence of victimization (34.81%) was reported more compared to the perpetration (23%). Further, in terms of mutual violence (being involved in victimization and perpetration) the data revealed that 13.71% of participants were in mutually psychologically aggressive relationships, whereas less than 1% of participants had experienced mutual sexual coercion or injury.

### 3.2. Bivariate Correlations

As the first step, zero-order correlations for all the examined variables were analyzed and are shown in Table 2. The duration of the intimate relationship was significantly positively correlated with perpetration of psychological aggression. Satisfaction within the relationship was negatively correlated with both types of psychological aggression (perpetration and victimization), and with injury caused by the respondent (perpetration). In terms of the facets of impulsivity, positive and negative urgency were positively correlated with victimization and perpetration of psychological aggression. Further, positive urgency was in negative correlation with the variable of physical assault victimization, while negative urgency was in positive correlation with the variable of injury victimization. Of the other facets of impulsivity, only sensation seeking was associated with dating violence, and was negatively correlated with perpetration of sexual coercion. Scores on the empathy subscale of perspective taking negatively correlated with physical assault perpetration, on the subscale of empathic concern they were positively correlated with sexual coercion victimization, whereas on the personal distress subscale, they were positively correlated with victimization in the form of psychological aggression.

### 3.3. Multiple Regression

The results of the hierarchical regression analysis performed for the criterion of perpetration of psychological aggression showed that the first set of predictors can explain approximately 12.4% of the variance in the outcome variable. The variables significantly associated in the first step were age (β = −0.17, *t* = −3.73, *p* = 0.000), duration of relationship (β = 0.20, *t* = 4.06, *p* = 0.000), and satisfaction with the relationship (β = −0.29, *t* = −6.39, *p* = 0.000). Impulsivity, used additionally in the second step of the analysis, explained 17.2% of the variance in perpetration of psychological aggression. In the second step, the variables of age (β = −0.17, *t* = −3.55, *p* = 0.000), duration of relationship (β = 0.22, *t* = 4.45, *p* = 0.000), relationship satisfaction (β = −0.26, *t* = −5.47, *p* = 0.000), and negative urgency (β = 0.26, *t* = 4.53, *p* = 0.000) proved to be significant predictors. The full and final set of variables for perpetration of psychological aggression explained 18.2% of the variance in the outcome variable. The significantly associated variables in the final step were age (β = −0.17, *t* = −3.47, *p* = 0.001), duration of relationship (β = 0.21, *t* = 4.28, *p* = 0.000), relationship satisfaction (β = −0.26, *t* = −5.51, *p* = 0.000), and negative urgency (β = 0.26, *t* = 4.43, *p* = 0.000). Further, for the physical assault perpetration criterion, the significant predictor “lack of perseverance”, entered in the second (β = 0.12, *t* = 2.03, *p* = 0.043) and final steps (β = 0.12, *t* = 2.03, *p* = 0.043), was found to be related to the criterion variable. Analysis of the criterion of perpetration of sexual coercion indicated that the set of predictor variables described can explain approximately 2.4% of the total variance of sexual coercion. After testing the possible contribution of the sociodemographic variables in the first step, which explained an insignificant 00.1% of the result on the sexual coercion subscale, the addition of the impulsivity facets, more precisely sensation seeking (β = −0.12, *t* = −2.21, *p* = 0.028), was able to explain an additional 1.7% of the variance. In the final model for perpetration of sexual coercion, sensation seeking (β = −0.13, *t* = −2.36, *p* = 0.019) was a unique predictor. Analysis of the explanation of injury perpetration showed that the variable of satisfaction with the relationship (β = −0.11, *t* = −2.20, *p* = 0.028) was a significant predictor. After introducing impulsivity variables in the second step, satisfaction with the relationship (β = −0.10, *t* = −2.04, *p* = 0.042) again remained the only significant predictor. In the final set of variables, which accounted for 2.2% of the variance in the outcome variables, the only unique predictor was satisfaction with the relationship (β = −0.11, *t* = −2.20, *p* = 0.028).

The results for psychological aggression victimization showed that in the first step, age (β = −0.15, *t* = −2.88, *p* = 0.004), duration of relationship (β = 0.13, *t* = 2.58, *p* = 0.010), and relationship satisfaction (β = −0.24, *t* = −5.27, *p* = 0.000) were the most significant predictors. The first set of variables explained 8.1% of the variance of psychological aggression victimization. In the second step, the introduction of the impulsivity resulted in an explanation of an additional 3.8% of variance. All the variables that were found to be significant in the first step continued to be significant in the second step (age: β = −0.14, *t* = −2.67, *p* = 0.008; duration of relationship: β = 0.15, *t* = 2.85, *p* = 0.005; relationship satisfaction: β = −0.21, *t* = −4.40, *p* = 0.000). It is important to note that in this step of analysis, the negative urgency was also a significant predictor (β = 0.23, *t* = 4.01, *p* = 0.000). In the third step, the percentage of the explained variance increased by 2.1% and all sociodemographic variables (age: β = −0.12, *t* = −2.32, *p* = 0.021; duration of relationship: β = 0.14, *t* = 2.75, *p* = 0.006; relationship satisfaction: β = −0.23, *t* = −4.66, *p* = 0.000), negative urgency (β = 0.22, *t* = 3.54, *p* = 0.000) and the perspective taking variable (β = 0.14, *t* = 2.55, *p* = 0.011) proved to be significant unique predictors. As for physical assault victimization as a criterion variable, the socio−demographic variables entered in the first step were not found to contribute to an explanation of the outcome variable. In the second step, a significant predictor was found to be lack of premeditation (β = 0.15, *t* = 2.45, *p* = 0.015), which accounted for 3.4% of the variance. The final, full set of variables in the regression of physical assault victimization explained 3.9% of the variance. The only unique was found to be lack of premeditation (β = 0.15, *t* = 2.33, *p* = 0.020). The results for sexual coercion victimization as a criterion variable were only significant in the last step, with empathic concern as a unique significant predictor (β = 0.15, *t* = 2.59, *p* = 0.010), explaining 2.6% of the variance. Finally, analysis for the injury victimization variable found that negative urgency in the second (β = 0.21, *t* = 3.50, *p* = 0.001) and third models was a significant predictor (β = 0.21, *t* = 3.24, *p* = 0.001). The results of regression analysis are presented in Table 3.

## 4. Discussion

According to the results of this study conducted among young college women, 57.81% reported engaging in at least one type of violent dating behavior in the previous year. The prevalence was similar to findings in a previous study conducted. According to a systematic review by Jennings et al. [2], the incidence of DV is between 9% and 37% among women. As their results were slightly lower than those observed in our study, the reason for this could be the differences in the operationalization of violence, in the measurement instruments, and in the different sample used, as well as methodological strategies of analysis [6].

Exposure to some types of DV in our study varied in terms of the type of violence. Experience of psychological aggression was more frequent than experience of injury, sexual coercion, or physical assault by a partner. Other studies have shown a similar prevalence for psychological violence [66,67,68], between 30% and 92%. In contrast to the results of our research, some other studies have found a higher prevalence for physical DV, showing that between 10% and 25% of women have been exposed to this kind of violence [1,69]. Studies measuring only sexual violence found that its prevalence among women ranged from 9% to 13% [70,71]. One possible explanation for this is that our participants may have underreported their experience. Underreporting may be associated with the stigma of being a victim, which has been widely present among adults [72,73]. In addition, it is perhaps easier for participants in research to report psychological violence than more severe violence, such as physical injuries or sexual violence. Finally, according to Hickman et al. [74], studies examining the overall prevalence often share similarities between the samples used, but produce different estimates.

In relation to perpetration of violence, psychological aggression appeared to be the most prevalent type in our study (23%). Physical assault was reported by 2.11% of the women, whereas occurrence of violent behavior causing injury and sexual coercion were reported by 1.48% and 0.84% participants, respectively. The occurrence of victimization (39.02%) was reported more than perpetration of violence (27.43%). Many researchers [75,76] noted that it is difficult to assess violence, especially for women who find it difficult to respond adequately to initial acts of violence against a partner. Moreover, studies show that an individual’s ability for labeling or recognizing experiences of violence as abuse is determined by several factors, such as: their attitude towards violence [77], experiences of childhood abuse [78], feelings of guilt [79], the victim’s feelings of fear [80], and defense mechanisms that minimize and justify violent behaviors with the purpose of protecting the positive aspects of the relationship [81].

The duration of the intimate relationship was positively correlated with perpetration of psychological aggression, according to the results of our study. A large amount of data suggest that violence is more likely to occur within more serious dating relationships [82,83] and in relationships of longer duration [84,85]. Longer duration can result in greater investment and commitment in relationships, but also may provide more opportunities for conflict to arise [86]. The duration of the relationship and engaging in sexual intercourse, according to Vivolo-Kantor et al. [85], were factors that could influence the style of relationship and communication [85]. Also, male-perpetrated DV is more acceptable within the context of a serious, committed relationship than within a less serious relationship [86,87].

Relationship satisfaction usually refers to the degree to which an individual feels positive about their partner and their relationship [88]. In our study it was negatively correlated with both types of psychological aggression (perpetration and victimization) and with injury caused by the respondent (perpetration). Our findings are in concordance with previous research, which found that victims are less satisfied with their intimate relationship [52,53,54]. Higher levels of DV have been linked with lower levels of victim satisfaction with their intimate relationship [89,90]. However, these studies focused primarily on women. Some other studies, conducted only on women, also found that women victims have a lower level of satisfaction with their relationship than women who are not victims of DV [91,92]. Our results can be explained by the assumptions of rewards and costs postulated by interdependence and social exchange theory. Using this theoretical approach, we can assume that violence between partners is perceived as a significant cost of being in the relationship, and can have a negative influence on relationship satisfaction for the victimized partner [52,89,90,93,94]. In addition, when violence occurs in a serious relationship that is based on love and trust, the impact on relationship satisfaction is likely to be much more damaging. As such, people in more committed, violent relationships may display lower relationship satisfaction than people in less committed relationships. Further, the relationship between DV perpetration and relationship satisfaction has not been studied extensively. Ulloa and Hammet [95] conducted a study on American women, aged 18–35, and found that women who commit violence perceive their relationships as less stable and are less happy than women who do not commit violence. Interestingly, Llorens et al. [96], in a longitudinal survey of young adults aged from 18 to 28 years, revealed a positive relationship between women’s IPV perpetration and relationship satisfaction. One of the explanations that researchers offered emphasized the perpetrators’ stronger feelings of power over the violence that occurs in the relationship. Our results, which indicated the negative association of relationship satisfaction with psychological and physical injury in relation to DV perpetration, could be explained in two ways. Relationship dissatisfaction may be a trigger for DV perpetration. Individuals dissatisfied with a relationship experience higher levels of negative emotions and may be more likely to respond to their partner with violence. It could also be the case that violent behavior may in turn increase the likelihood of relationship distress and less satisfaction. However, more research in this field is clearly warranted.

We were interested in examining whether levels of impulsivity are in correlation with DV victimization and perpetration. According to the results, positive urgency was positively linked with victimization and perpetration of psychological aggression. Negative urgency was positively linked with injury victimization. From our results, we can conclude that impulsive people, when they are in a positive mood, are prone to be involved in DV, as a victim or perpetrator. When they are in a negative mood, they are more prone to use physical aggression. What we know so far is that individuals who are high in impulsivity engage in impulsive behaviors in an effort to reduce their negative emotions, without considering the consequences of these behaviors. It has been suggested that impulsivity is especially deleterious because, when faced with distressing situations, impulsive individuals are more likely to utilize the most easily available methods for coping in order to obtain short-term relief, despite the potential long-term consequences, which are negative [47,97]. Helfritz and Stanford [98] argued that impulsivity can be a basis for a variety of disorders and behaviors that are associated with irritability, low emotional stability, and uncontrollability. If this is true, then there may be more aggression related to impulsivity in the college setting, where students are usually struggling with various dating relationships and peer pressure, and may find themselves in emotionally affective situations that increase the likelihood of violence (e.g., partner abuse). In addition, aggression will mainly occur during adolescence and adulthood since the development of the prefrontal cortex is not complete until the late 20s or early 30s, which may lead to difficulties with inhibitory control and emotion regulation, as well as fear conditioning [99].

Echeburúa and Fernández [100] stated that decreased empathy has especially negative impacts when manifested in an intimate sphere, where it is important to show trust, support, and emotional connection. According to the results of our study, the tendency to accept the psychological views of others spontaneously is negatively correlated with perpetration in dating relationships, specifically with physical aggression. Empirical studies of empathy in perpetrators of DV are relatively limited in number, and there are even fewer studies on victims. As a result, we will also compare our results with the results of studies in the field of IPV. Our results are in concordance with the results of Covell et al. [55], who conducted research on male perpetrators. They also used Davis’ [63] concept of empathy and noted that there are several different profiles of perpetrators associated with different types of violence, although perspective taking was related to all types of violence. Péloquin et al. [56] examined romantic attachment, dyadic empathy, and psychological partner aggression in 193 couples recruited from the community who had been cohabiting for at least six months. Their mean age was 31 years, and the majority (60%) had a college degree. The results showed that, in women, decreased empathy was associated with increased psychological aggression.

If we analyze the results for victimization, empathic concern was positively correlated with sexual coercion, and the results on the personal distress scale were positively correlated with psychological aggression. Our results are different from the results of Péloquin et al. [56] who concluded that low empathy in women is associated with victimization. The positive correlation between high empathy (specifically the tendency to experience feelings of sympathy, concern, and personal anxiety in tense interpersonal settings) and victimization in our study could be explained by the young women’s understanding of their partners’ frustration in interpersonal conflicts. Understanding of the perpetrator’s perspective can contribute to the victim continuing to be in the violent relationship. Nonetheless, the study comprised a preliminary investigation of empathy in young women in abusive dating relationships, and suggests empathy is a construct that merits further investigation in studies of DV. In this context, consideration of other empathy measures is also desirable. Namely, although numerous studies indicated the validity and reliability of using Davis’s multidimensional measure [29,101], further examination of its different aspects of validity (e.g., convergent validity, discriminant validity) based more on a theoretical and empirical approach is needed.

We found that relationship duration and relationship satisfaction are predictors of psychological aggression perpetration, while relationship satisfaction is a predictor of injury perpetration. Our results confirm the results of previous research [26,84,85]. We can conclude that if we want to understand the dynamics of DV, we have to pay more attention to long-lasting relationships and the quality of relationships, specifically the relationship satisfaction. The evidence suggests that in young adulthood, intimate relationships become longer and of better quality. As young adults develop better relationships, they become more and more distant from abusive behaviors [102].

In our study, measures of impulsivity (negative urgency, sensation seeking, and lack of perseveration) significantly predicted perpetration of psychological aggression, physical assault perpetration, and sexual coercion. A relationship between impulsivity and IPV has consistently been found in the literature, which suggests that impulsivity contributes to violence [103,104]. We expected that impulsive behavior would increase the likelihood of aggressive behavior among young women. It is assumed that many perpetrators act impulsively, ignoring the consequences of their violent behavior. In addition, expression of aggression associated with impulsivity may increase in a college environment, where students typically struggle with a variety of dating relationships and peer pressure, and may find themselves in emotionally affective situations that increase the likelihood of violence (e.g., partner abuse). Some authors have suggested that in modern Western society, this development period is a period of prolonged adolescence, sometimes referred to as “emerging adulthood” [105], which may provide insight into the potentially proactive type of aggressive behavior. Moffitt [106] suggested that adolescents experience a maturity gap, where they want to have the benefits of being adult but are not yet able to reach them. It is then possible for some young adults to turn to aggressive behavior to achieve their desired resources, power, or sex. Further, we must point that low self-control and experiences of heightened arousal can be the results of neurological development. From adolescence to early adulthood, changes in the prefrontal cortex occur [107]—the region that is important for control of various aspects of behavior [108].

Our study showed that impulsivity (lack of perseverance and negative urgency) do not just predict DV perpetration, but also victimization (injury victimization and psychological aggression victimization). More research is needed in this field to understand the role of impulsivity in DV victimization and perpetration.

Finally, we found that empathic concern and perspective taking are significant predictors of sexual coercion and psychological aggression victimization. Empathy is thought to have an inhibitory influence on an individual’s tendency to behave aggressively due to the cognitive dissonance experienced [109], which, in turn, contributes to a decrease in the risk for violence [110].

As far as we know, no previous studies have analyzed the relationship between empathy and specific type of DV. Previous research has, however, supported the view that DV perpetrators have greater deficits in perspective taking and emotional decoding processes than controls [111]. In line with this, deficits in processes for decoding emotional stimuli, especially those with a neutral value, have been shown to explain why they have a poorer capacity to understand the thoughts and emotions of others (cognitive empathy and perspective taking) than nonviolent men [111,112]. Weak cognitive empathy could explain why aggressors misunderstand and attribute hostile connotations to neutral stimuli, increasing the likelihood of them behaving aggressively [113], and the high rates of recidivism after specific aggressor treatment [114]. Taken together, it seems essential for empathy to be considered in intimate partner violence intervention and prevention programs designed for perpetrators in intimate relationship, precisely those that employ psychotherapy. One of the focuses in treatment plans should be improvement of their empathic skills. Romero Martinez, Lila, and Moya Albiol [115] stated that after intervention programs for DV perpetrators, practitioners must check whether they have better empathy skills. Besides that, it is important to follow up with perpetrators after the program to check how any changes in these skills affect their risk of recidivism.

## 5. Limitations

The limitations in our research are mainly related to its retrospective methodological approach. The information requested from participants is of an intimate nature and requires them to recall negative life experiences. Some experiences could have been suppressed and forgotten, while others could be exaggerated. Their recollections may have evoked various emotions that could have affected the participants’ ability to give a critical evaluation of their partners’ behavior. The sample comprised young college women representing a single geographical area. Also, longitudinal research would allow examination of the causal relationship between the variables. The sample was recruited online, so participants were self-selected. Furthermore, the participants were only young women. The young college men did not accept the invitation to participate in the research.

## 6. Conclusions

Bearing the limitations discussed above in mind, it may be concluded from the present study: (1) that victimization that includes psychological aggression is more frequent than perpetration of DV among female college women; (2) that the impact of perpetration and victimization might differ in relation to age, relationship satisfaction, and individual factors—empathy and impulsivity. Younger adults and those in a longer relationship, as well as those dissatisfied with their relationship, are more prone to DV. Impulsivity facets, especially those related to a lack of perseverance and negative urgency, have significance in explaining DV experience and perpetration. Empathy related to perspective taking or empathic concern are important in the prediction of psychological and sexual violence In conclusion, this study offers an initial consideration of the prevalence of violence among female college students, and an insight into the relationship between the demographic and individual risk factors for DV perpetration and victimization, which may be relevant for researchers, as well as practitioners involved in treatment of violent relationships between young people. Future prevention and intervention programs should take into account the complex pattern of relationships between DV and demographic and individual factors. Professionals should pay attention to age differences and the duration and quality of the relationship, as well as focusing on developing self-regulation and empathy skills among those exposed to DV.

## Figures and Tables

**Table 1:** Percentage of participants who reported dating violence.

Dating Violence	Perpetration Only	Victimization Only	Mutual Violence	Total Violence
	N (%)	N (%)	N (%)	N (%)
Psychological aggression	109 (23)	165 (34.81)	65 (13.71)	274 (57.81)
Physical assault	10 (2.11)	2 (0.42)	0 (0)	12 (2.53)
Sexual coercion	4 (0.84)	4 (0.84)	1 (0.21)	8 (1.69)
Injury	7 (1.48)	14 (2.95)	4 (0.84)	21 (4.43)

**Table 2:** Intercorrelation of all predictor variables with dating perpetration and victimization.

	Perpetration				Victimization			
Variables	Psychological	Physical	Sexual	Injury	Psychological	Physical	Sexual	Injury
Age	0.09	−0.02	−0.02	−0.00	0.07	0.06	−0.00	0.07
Duration of relationship	0.13 **	−0.06	0.02	−0.04	0.08	−0.02	0.02	0.04
Satisfaction with relationship	−0.26 ***	−0.01	0.03	−0.09 *	−0.23 ***	0.02	−0.02	−0.04
Negative urgency	0.26 ***	0.05	−0.04	0.06	0.23 ***	−0.09	0.02	0.13 **
Lack of perseverance	0.04	0.06	−0.02	0.01	0.04	−0.00	−0.04	0.01
Lack of premeditation	0.07	0.01	−0.02	−0.02	0.08	0.06	−0.02	0.04
Sensation seeking	0.08	0.01	−0.11 *	0.03	0.05	−0.08	0.00	−0.02
Positive urgency	0.12 **	0.05	−0.01	0.03	0.10 *	−0.11 *	−0.04	0.01
Perspective taking	−0.00	−0.10 *	−0.05	−0.02	0.05	−0.00	−0.02	−0.06
Fantasy	0.07	0.00	−0.00	−0.03	0.08	−0.08	−0.00	0.05
Empathic concern	0.09	−0.08	−0.03	−0.02	0.06	−0.05	0.11 *	0.02
Personal distress	0.09	−0.01	−0.03	0.00	0.14 **	−0.08	0.05	0.06

Note: Psychological = psychological aggression; physical = physical assault; sexual = sexual coercion; * *p* < 0.05. ** *p* < 0.01. *** *p* < 0.001.

**Table 3:** Multiple regression analysis for experience of dating violence by socio−demographic variables, impulsivity and empathy.

	Perpetration			Victimization		
Variables	1st Step	2nd Step	3rd Step	1st Step	2nd Step	3rd Step
Psychological aggression						
R^2^	0.12	0.17	0.18	0.08	0.12	0.14
Adjusted R^2^	0.12	0.16	0.16	0.07	0.10	0.16
R^2^ change	0.12	0.05	0.01	0.08	0.04	0.02
F	19.75 ***	10.72 ***	7.57 ***	12.21 ***	6.92 ***	5.52 ***
Significant F change	0.00	0.00	0.30	0.00	0.00	0.04
Physical assault						
R^2^	0.07	0.02	0.03	0.01	0.03	0.04
Adjusted R^2^	−0.00	−0.00	0.00	−0.00	0.02	0.01
R^2^ change	0.01	0.01	0.01	0.001	0.03	0.01
F	0.83	0.93	1.02	0.66	1.79	1.38
Significant F change	0.48	0.42	0.31	0.58	0.03	0.68
Sexual coercion						
R^2^	0.00	0.02	0.02	0.00	0.01	0.03
Adjusted R^2^	−0.01	−0.00	−0.01	−0.01	−0.01	−0.00
R^2^ change	0.00	0.02	0.01	0.00	0.01	0.02
F	0.18	0.89	0.84	0.13	0.36	0.92
Significant F change	0.91	0.26	0.56	0.95	0.78	0.09
Injury						
R^2^	0.01	0.02	0.02	0.01	0.04	0.04
Adjusted R^2^	0.01	−0.00	−0.01	−0.00	0.02	0.02
R^2^ change	0.01	0.00	0.00	0.01	0.03	0.01
F	1.92	0.95	0.77	0.92	2.08 *	1.58
Significant F change	0.13	0.87	0.80	0.43	0.02	0.65

Note: * *p* < 0.05; *** *p* < 0.001.
